# Humanized Mice as a Tool to Study Sepsis—More Than Meets the Eye

**DOI:** 10.3390/ijms22052403

**Published:** 2021-02-27

**Authors:** Krzysztof Laudanski

**Affiliations:** 1 Department of Anesthesiology and Critical Care, University of Pennsylvania, Philadelphia, PA 19194, USA; klaudanski@gmail.com; Tel.: +1-215-662-8000; 2 Department of Neurology, University of Pennsylvania, Philadelphia, PA 19194, USA; 3 Leonard Davis Institute of Healthcare Economics, University of Pennsylvania, Philadelphia, PA 19194, USA

**Keywords:** sepsis, septic shock, stem cells, humanized mice, methods, animal models

## Abstract

(1) Background. Repetitive animal studies that have disappointed upon translation into clinical therapies have led to an increased appreciation of humanized mice as a remedy to the shortcomings of rodent-based models. However, their limitations have to be understood in depth. (2) Methods. This is a narrative, comprehensive review of humanized mice and sepsis literature to understand the model’s benefits and shortcomings. (3) Results: Studies involving humanized models of sepsis include bacterial, viral, and protozoan etiology. Humanized mice provided several unique insights into the etiology and natural history of sepsis and are particularly useful in studying Ebola, and certain viral and protozoan infections. However, studies are relatively sparse and based on several different models of sepsis and humanized animals. (4) Conclusions. The utilization of humanized mice as a model for sepsis presents complex limitations that, once surpassed, hold some potential for the advancement of sepsis etiology and treatment.

## 1. Introduction

Sepsis, septic shock, septicemia, or the more colloquial blood poisoning, is a prevalent, mortal, and formidable challenge in medicine [1]. In a recent consensus statement, sepsis was defined as a “*life-threatening organ dysfunction caused by the dysregulated host response to infection*,” but even this definition is challenged [2,3]. So far, the definition of sepsis predominantly relies on physicians’ judgment compounded with vague and ambiguous symptomology. The pathological processes associated with sepsis are most likely very heterogeneous, however this is rarely appreciated in clinical settings [4]. Sepsis is often linked to bacterial triggers, however any pathogen can elicit the immune system pathological response [5]. Sepsis has a tremendous impact in developed, developing, and austere countries. In the United States alone, the incidence of sepsis encompasses 6% of all hospital admissions. The mortality varies between 20% and 40% [1,4,6]. The costs related to sepsis treatment and subsequent disability of survivors is 20 billion dollars per annum in the United States alone, with profound and difficult-to-quantify individual and societal consequences [6,7,8]. Only early engagement of antibiotic was demonstrated to improve sepsis outcome [4,6]. Significant efforts were directed towards finding effective remedies for sepsis, but, almost universally, they failed in terms of the translation of discoveries in animal models to clinical practice [9,10].

### 1.1. General Description of Septic Response

Sepsis is initiated by binding of the pathogen-associated molecular pattern (PAMP) to toll-like receptors (TLR) followed by the activation of innate immunity [4,6]. This early response activates the endothelium, complements coagulation, lactoferrin, transferrin, NK cells, phagocytes, and neutrophils, as well as a variety of other mechanisms. Concomitantly, neuronal inflammatory reflexes augment immune system responses [11]. Cytokine release induces several symptoms including fever (IL-6, prostaglandins), switch to catabolism (TNFα, IL-6), tachycardia and tachypnea. Vasodilation and increased vascular permeability leads to tissue swelling, hypoxia, and intravascular volume depletion [12]. Ensuing hypotension is compounded by septic cardiomyopathy [13]. Oxygen delivery to the organs may be further impaired by microcirculation defects, increased endothelial permeability, and mitochondrial abnormalities [6,12,14,15,16]. The collateral damage due to innate immunity activation is mediated by the nonspecific nature of endogenous bacteriocidic agents, innate effects of IL-1β, TNFα, granzymes, or prostaglandin metabolism products. Activation of the coagulation cascade may result in disseminated intravascular coagulation [17]. As the innate system response peaks, monocytes, dendritic cells, and, to some degree B cells, capture and process antigens [4]. Subsequently, specific T and B clones undergo a massive buildup, directing the immune response precisely against the invading pathogen and limiting collateral damage. Upon eradication of the pathogen, a de-activation of the immune system has to occur. This part of the process is called compensatory anti-inflammatory response syndrome (CARS), but the name implies that the immune system is actively suppressed [4,18]. In fact, during CARS the immune system is being de-activated, not suppressed [4].

This intricate process can be derailed in several ways, leading to different sepsis presentations [6,9,18]. More importantly, the complex nature of sepsis is species-specific, putting the utility of the rodent models of sepsis to question [19]. Here, we provide a narrative review of research involving humanized mice with special emphasis on their benefits and shortcomings.

### 1.2. Models of Sepsis in Small Animals

**Figure 1:**
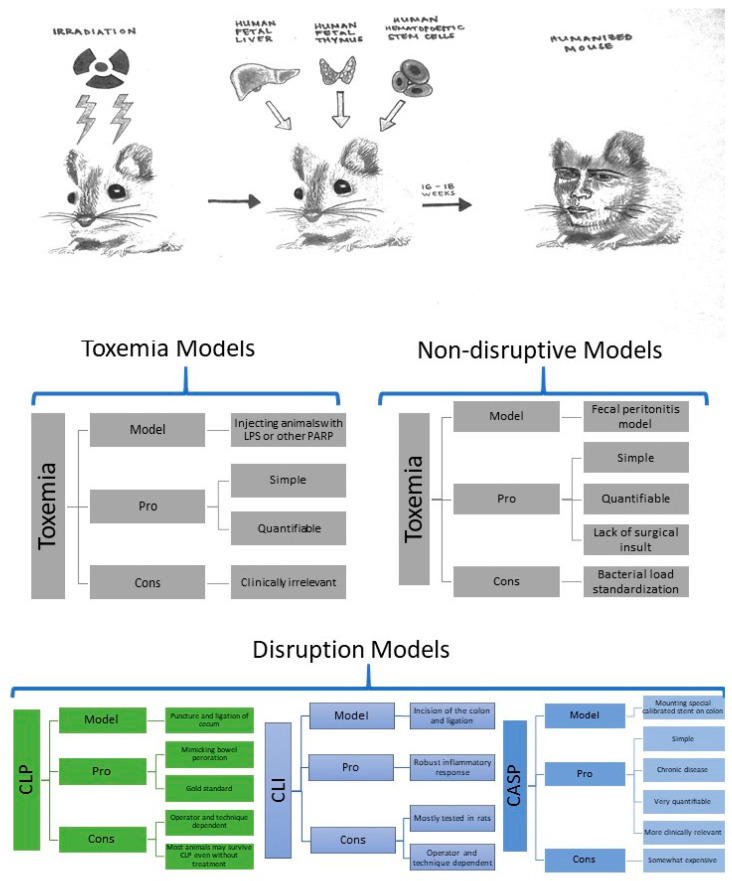
Animals models of septic shock. LPS – lipopolysaccharide, PARP – pathogens associated recognition patterns, CLP – cecal ligation and puncture, CLI – cecal ligation and incision, CASP - colon ascendants stent peritonitis

There are three main approaches to model sepsis on rodents (Figure 1).

The most straightforward approach to mimic sepsis is the toxemia model. Lipopolysaccharide (LPS), or another PAMP, is injected into animals to trigger an immune response [20,21,22,23,24]. This allows for high standardization and reproducibility, but the model is so distant from clinical relevance that it should not be referred to as a septic model. However, it is useful to study essential immune responses allowing for precise manipulation of the kind and dose of pathogen used. Type of infected compartment of infection and host susceptibility are essential factors determining animal responses in the toxemia model [25,26].

Host disruption models of sepsis approximate clinical sepsis with greater accuracy. They include cecal ligation and puncture (CLP), cecal ligation and incision (CLI), and colon ascendants stent peritonitis (CASP) [27,28,29]. All these models disrupt the gut barrier, allowing colon flora to induce sepsis-like immune responses [30]. Their advantages and shortcomings are summarized in Figure 1.

The CLP model including humanized mice has been utilized frequently [31,32,33]. One study compared both LPS-endotoxemia and the CLP model [33]. The bone marrow progenitor changes were distinct in these two models, suggesting that CLP and endotoxemia models are not equivalent. Besides problems with model equivalency and variable mortality, CLP and CLI models are susceptible to surgical conditions, making it challenging to compare studies published in different labs.

The fecal slurry model is the only model where there is no disruption of the gut barrier [34]. The fecal matter is given directly to the peritoneum. Variability in this model stems from different qualitative and quantitative differences in fecal matter. Advantages are the lack of the surgical injury to animal. The authors did not find a single case of use of this model in humanized animals

### 1.3. The Need for Humanized Mice Sepsis Research

Several reasons underlying unsuccessful translation of the animal studies in therapies were articulated [27,35,36]. Mice and rats have a significantly higher resistance to sepsis as compared to humans. Certain animals are inbred and usually at the same age, resulting in uniform response with loss of sepsis presentation heterogeneity. The interactions between key internal organs (neuronal, immune systems, others) may be different in rodents, further limiting their equivalency [11]. Pre-existing co-morbidities are infrequently present in animal models before inducing sepsis, but they are important factors in human sepsis [35,37]. After the induction of sepsis, a minority of researchers treat animals in a way resembling the clinical treatment provided to septic patients, because most studies are short term [10,28,30,38]. Additionally, several manifestations of organ failures have no clear equivalences in rodents and humans (e.g., mental status). Neither are immune system components identical in both species, with the spleen being a prime example of an organ with a distinct role in the human vs mice immune system [39,40]. Several of these concerns were raised in “Of mice, not a man”, a landmark opinion piece upsetting long established beliefs, paradigms and culture in sepsis research [35]. Subsequent discussion about a relevance of animal models to human sepsis remains rather animated, but it is difficult to deny that mice immune systems have several distinctive features [1,4,35,36,38].

### 1.4. Introduction to Models of Humanized Mice

Humanized mice are xenotransplant animals with a human immune system developed through the expansion of grafted human cells [41]. Subsequent models of immune system reconstitution approximate the human immune system with increased accuracy while depressing the native immune system more completely.

The creation of the humanized mice starts from a mouse strain with acquired immunodeficiency [41]. The most common type used is SCID [41,42,43,44,45]. Other strains frequently used are IL-2^rgnull^, NOD.Cg-Prkdc^scid^Il2^rgtm1Wjl^ (NSG), NODShi.Cg-Prkdc^scid^Il2rg^tm1Sug^ (NOG), and C;129S4- Rag2^tm1Flv^Il2rg^tm1Flv^ (BRG) mice [46]. All these strains suffer significant immune-system deficits, allowing for more efficient grafting and reducing the emergence of graft vs host disease [45,47,48]. However, animals have to be irradiated before grafting to suppress the native immune system.

Once the mice are deprived of their native immune system, the implantation of the human immune system can occur. Depending on the type of transplanted tissue, three major types of humanized mice exist (Table 1).

Finally, humanized animals may be genetically modified to provide them with the endogenous ability to produce human cytokines (GM-CSF, Flt-L) to support the growth and survival of the implanted human immune system [49].

## 2. Overview of Utility of Using Humanized Mice in Sepsis

### 2.1. Bacterial Sepsis

The septic shock and humanized mice narrative began from a groundbreaking study by Hotchkiss et al., utilizing CLP to induce sepsis and euthanizing animals after 24 h [50]. They demonstrated that the production of cytokines in response to infectious insult was significantly increased in animals 20 weeks after grafting. Most importantly, humanized mice had a profound apoptotic response, a commonplace phenomenon in the aftermath of sepsis [4]. Additionally, delayed hypersensitivity skin reaction was diminished. However, animals were euthanized 24 h after sepsis induction, limiting the study to the acute response phase. Mortality was unknown among the cohort of CLP induced animals. Animals did not receive fluid resuscitation or antibiotics. Despite Hotchkiss’s pioneering research, it is still surprising that it took over 15 years for the first study of septic shock in humanized mice to appear since the first introduction of this model in 1992 [26]. Further studies confirmed the secretion of TNFα, IL-6, and IL-10 in response to TLR-stimulation by PAMP in humanized mice [21,51]. Concomitantly, the release of high mobility group box 1 (HMGB1) in sepsis was demonstrated and established a viable therapeutic option six years before the first similar human study [52,53]. Inflammasome complexes (NFL3) were demonstrated in humanized mice, but in a model of autoimmune arthritis [54].

Several other bacterial diseases were demonstrated in humanized mice. Typhoid infection in the SCID humanized mice replicated the entire disease process [55]. Skin infection with Staphylococcus in humanized mice showed a clinical resemblance, however animals required a higher dose of inoculated bacteria while subsequent lesions were more pronounced than wild-type animals [56]. Humanized mice were instrumental in discovering a novel way of interaction of Staphylococcus Enterotoxin B (SEB) with iNKT or MAIT cells [20,22]. The same group translated these results to mouse models, establishing several important links [23,57]. Knop et al. utilized humanized mice to simulate Gram-positive bacterial infection instead of SEB toxemia. They demonstrated several common responses typical of sepsis as in classic study by Hotchkiss’s group [50,58]. Most importantly, they compared the responses from humanized mice to wild type. Humanized mice had higher weight loss over five days after CLP induction, validating their translational relevance, because cachexia is sign of sepsis. Above all, they showed emergence of T cell anergy and Fas expression as compared to the wild type. These are human-specific responses to the septic challenge [4]. In a separate study, BLT-type humanized mice were used as a model for sepsis with a 28 day euthanasia window. Mice were treated with antibiotics and fluid resuscitation, however the mortality was a significant problem and required modification of the protocol [31,32]. The study demonstrated a change in human monocyte and dendritic cells’ reactiveness in animals surviving sepsis after 28 days from the original insult. M-CSF production secondary to PU.1 epigenetic dysregulation was one of the main mechanisms driving the abnormal fate of MO [31]. This finding is well-aligned with the belief that epigenetic changes sustained sepsis-induced changes long after the septic episode was resolved [59]. Transplant of stem cells partially restored the pre-septic function of the immune system, but the underlying mechanism was somewhat unclear. Bone marrow dysfunction was frequently suggested as a primary driver in immune suppression in sepsis [4,60]. Skirecki et al. showed early impairment of bone marrow progenitors in a humanized mouse model of CLP sepsis [33]. However, the relatively short time between re-grafting and sacrificing animals could preclude full restoration of the immune system.

Humanized mice were utilized to model neonatal sepsis in two studies from the same research group to demonstrate potential effect of prostaglandin and steroid modulators [25,26]. Pervasively, authors argued that imperfection of the human immune system in humanized mice equates to the “immature” immune system seen in neonates. However, neonates’ immune systems are relatively well developed, and neonatal sepsis has several distinctive features [61]. Endogenous immune system dysfunctions in humanized mice result from imperfection in several immunological compartments, co-existence with the mouse immune system, and scarcity of human growth factors, not neonatal-like immaturity [62,63]. It is far-reaching to equate the imperfection of the immune system performance related to the xenotransplant nature of the model as an indication that this resembles neonatal immune system features.

The emergence of anergy or increased prevalence of monocyte suppressive cells were observed during septic shock in humanized mice and wild animals [20,57]. Both mechanisms are part of de-escalation mechanisms, and their emergence is a complex process during the evolution of the response to septic shock [4]. Emergence of unfavorable T cell anergy was signified by increasing the expression of TIM-3, LAG-3, PD-1L, and PD-1 in humanized mice [64].

### 2.2. Viral Sepsis

Modeling HIV infection is one of the success stories for humanized mice, which has provided a sustainable model to study infection and a platform for testing several HIV/AIDS-related phenomenon [65,66,67,68,69]. Zika infection and related viremia have also been well studied in humanized models [70]. Dengue infection is almost universally complicated by hemorrhagic fever and death, and humanized mice are a perfect model to study this, considering the high mortality of these diseases [71,72]. The pivotal role of NK and IFNγ in protecting against acute viremia in hemorrhagic fever was discovered using this model [73].

Dobner et al. showed that NSG humanized mice could reproduce acute and chronic adenoviral infection [74]. Most importantly, the persistence of chronic infection was signified by established immunity and expression of viral genes within bone marrow only. These findings aligned exceptionally well with clinical findings. The authors did not test the animals with sepsis models afterward to establish if the viral infection would re-activate, because this has been observed in some survivors of sepsis.

Similarly, Epstein–Barr virus (EBV) infection was successfully established in humanized mice [75,76]. In a more recent study, the authors suggested that EBV infection leads to hemophagocytic lymphohistiocytosis (HLH), but they failed to provide clinical data supporting such an assumption. HLH is increasingly appreciated as the illness producing a sepsis-like syndrome, and introduction of a humanized model could be instrumental in establishing effective therapies [77].

There is no report to date investigating the application of humanized mice to study SARS-CoV-2, except one review [5,78].

### 2.3. Protozoan Infection

The immune system response to protozoa is often overlooked, even though malaria is one of the world’s most prevalent conditions. Humanized mice can sustain the whole life cycle of *Plasmodium falciparum* if both human liver tissue and erythroid compartment have been provided [79,80,81,82,83]. Immune responses to malaria are different from sepsis in several aspects, but the dysregulated immunological response is commonplace among these two conditions [83]. Humanized mice allowed for the discovery of the exosomes in malaria, delivering proof of the principle regarding new diagnostic techniques [84]. New therapeutic compounds and vaccine testing were conducted using humanized mice as well [85,86].

## 3. Discussion

### 3.1. The Overall Advantage of Humanized Models of Sepsis

The impetus for the development of humanized mice was to understand sepsis pathophysiology, to accelerate the development of new therapies, to minimize exposure of human subjects to potentially ineffective or even harmful therapies, and to decrease commercialization costs for promising therapies [4,19,25,35,44,47]. The overall effort to produce, breed, and maintain these hybrid animals has been lower than conducting research with human subjects, but substantially higher than using a rodent sepsis model. The question arises if the animal models are worth the additional cost and effort.

Our review of the literature showed that humanized mice contributed to understanding sepsis. The highlights of utilizing humanize mice models are concentrated: on describing the mechanism of apoptosis, interactions of staphylococcal superantigens with the immune system, development of vasculitis in hemorrhagic fever and meningitis, and short- and long-term reprogramming of the leukocyte via epigenetic mechanisms [32,57,58,70,74,79,85,87]. Humanized mice enabled demonstration of the causative effect and producing experiments that could be replicated using virtually identical animals, re-created from the same stem cell batch.

On a few occasions, humanized animals were employed to test certain compounds’ effectiveness to modify sepsis’s natural history. Ernst et al. published two papers showing the effect of betamethasone and indomethacin in neonatal sepsis modeled by “immaturity” of the humanized mice immunity [25,26]. A third paper complemented these studies, showing a similar effect when a pathogen from Gram-positive bacteria stimulated neonatal cord mononuclear leukocytes [25,88]. They observed several parallel responses in both experiments, suggesting that betamethasone may be sufficient to moderate cytokine release in neonates compared to betamethasone with indomethacin [88]. The most significant contribution of the studies conducted by Ernst et al. is that they could prevent unnecessary testing on human subjects. Ye et al. tested HMGB1 siRNA using a short AchR binding peptide to mitigate cytokine storm in the CLP model of sepsis induced in humanized mice (BLT model) [52,89]. Humanized mice were used as a testbed for modulating the expression of PD-1, one of the most promising therapeutic agents, verifying several observations from human and animal models [64,87]. The C5aR antagonist was tested in a humanized model of Staphylococcal skin infection [56]. The drug reduced inflammation but resulted in immunosuppression that had an overall detrimental effect on survival. Utilization of the humanized models in chronic viral infection and malaria was credited with reduced harm, accelerated testing, and reduced costs [86,90]. Finally, autogeneic transplant of stem cells to the humanized BLT mice previously exposed to CLP sepsis seemed to correct peripheral and bone marrow immunological level properties, as was theorized previously [31,33,60]. Of note, transfer of mesenchymal cell was shown to reverse at least some detrimental effects of sepsis [91]. Consequently, testing the drug in humanized mice eliminated the risk in human subjects.

### 3.2. The Limitations of Humanized Models of Sepsis

Some limitations of humanized mice in the study of sepsis are related to animals’ use in general. There are some excellent reviews comparing human sepsis to murine models [35,36]. Here, we focus on limitations specific to humanized mice.

One of the primary deficiencies of humanized mice is a dynamic process of reconstitution of the human immune system in terms of the number of competent cells and composition of the immune system. Absence of one leukocyte population may hamper immune responses in general [92]. At the same time, the emerging human immune system must acquire a tolerance to the residual host’s immune system, dampening the graft versus the host disease, thus allowing native immune system to survive. Concomitantly, the transplanted immune system is immersed in mouse-originated growth [40]. Consequently, the reconstitution of human immune systems in xenotransplants is not absolute, but rather an ever-changing work in progress [42,62,63,92]. The development of more sophisticated humanized mice models (HIS, BLT) significantly reduced this problem, but did not eliminate it [38,41,47,66,70].

The second confounder of humanized mice is the fact that two immune systems co-exist. The positivity for CD45, a marker of successful grafting, varies between 20% and 80% [44,47,71]. Conversely, between 20% to 80% of the cells are mouse in origin. It is unclear what the remaining cells are functionally, or how they affect overall performance septic immune response. The level of grafting is very infrequently reported by researchers.

Upon successful grafting, two processes emerge simultaneously: (1) reconstitution of the components of the human immune system; and (2) resurgence of the native mouse immune system. Animals who were grafted with human stem cells needed time to reconstitute their human immune system, but at the same time, the strongly suppressed mouse immune system will attempt to reconstitute itself [21,42]. As the implanted stem cells age, the human immune system matures with different leukocyte compartments emerging at different times, but the risk of the resurgence of native immune systems is also higher in older animals. In more sophisticated models of humanized mice, an emergence of lymphoma due to the dysfunction of the native immune system was cited, but the introduction of double ablation methods alleviated this problem. Then, another study emerged suggesting higher mortality in humanized mice secondary to the inhibitory effect of mouse cells on human grafted leukocytes [51]. These interactions are difficult to measure, and account for, during cross-study comparisons. Both immune systems interact with each other, however there is almost no literature deep-diving into the importance of the process, especially in the context of time.

The innate nature of the laboratory animals does not mimic the human population’s heterogeneity, or the clinical reality experienced by patients. Humanized mice are even more inbred because their hosts are highly preselected. It is somewhat unclear if implantation of a series of homogenized animals with stem cells obtained from the same donor should be counted as separate animals or “clones”. This may present the future researcher with a thought-provoking dilemma. Should they use multiple stem cells to create several progenies? Should they treat animals grafted with the same stem cells longitudinally? Furthermore, there is randomness in how stem cells settle and develop in various leukocyte lineages, even in inbred animals [21,33,50,54,56,61]. However, such heterogeneity still does not reach the level of human heterogeneity.

Not a single manuscript involving humanized animals with pre-existing co-morbidity of some kind was identified by the author. Animals are kept in artificial, sterile, highly controlled, socially isolating conditions. Except for one study, no therapeutic intervention typically applied during sepsis was employed to mimic current states of disease.

Another question concerns the age of animals, especially if research focuses on age extremes. However, the question arises as to how we should account for the humanized mouse’s age. Is their age at the time of transplant the actual age of the mice or the stem cells’ age? In the study by Lange, animals used were of two “age” groups. Animals were subjected to resting 8–13 weeks versus 15–22 weeks after transplantation [21]. He suggested that cells after transplantation undergo maturation reflecting the aging of the immune system. Lange et al. arrived at a conclusion that seemed to juxtapose to findings presented by Ernst et al. [21,25,26]. The latter suggested that deficiencies of the humanized mice immune systems reflect neonatal immature immune systems [25,88]. Neither group of researchers provided sufficient evidence of the accurate and verifiable age of the humanized immune system. Furthermore, only 20% of cells were positive for the human CD45 antigen, suggesting that grafting was not completed, and some native immune systems survived. A more reasonable approach was shown by Andre, who grafted one group of mouse neonatal umbilical cord-blood derived cells, while another group was created used adult peripheral stem cells [62]. This demonstrated the emergence of impaired CD80^+^CD86^+^ cells and the reduced T cell ability to stimulate after the induction of sepsis in aged animals. The question of age is important because it influences several factors in the immune system response to pathogens, particularly the production of IL-6, a critical molecule in septic response [21]. These data suggests that the age of the grafted cells is a dominant factor in the aging of humanized mice, but it is unknown whether humanized leukocytes aged at the same speed as their “regular” counterparts.

Mouse host repopulation of the immune system typically occurs in the presence of human growth cytokines and hormones, but the bone marrow support system is of mouse origin [40,48,91,93]. Furthermore, the mesenchymal system is damaged by radiation used to suppress the mouse immune system before grafting. This creates a substandard cytokine environment for human stem cells due to the production of mice cytokines, a process which is further weakened by radiation injury. Newer models of humanized mice partially mitigate this problem by attempting complete reconstitution of the supportive environment in bone marrow, but entirely successful reconstitutions are not possible at this time, even with modern techniques supplementing human growth factors and cytokines [19,38]. There are several techniques to provide human cytokines to boost growth of the transplanted human immune system. Some of them rely on grafting additional human tissue such as the liver to study malaria, or blood vessels to investigate sepsis-related vasculitis [71,82,84,94,95,96]. Some studies supplemented human growth factors exogenously with the recovery of individual leukocyte compartments. Another approach is to genetically modify animals to provide more human cytokines and growth factors [97,98]. However, the introduction of several genes may skew the leukocyte population and potentially render the model questionable [97]. It is uncertain how these modifications affect the quality of the immune system and influence the results of the studies.

Crosstalk between different organs during sepsis is a critical element modulating the natural history of sepsis. The immunological nervous reflex is a significant contributor to the de-activation and moderation of the immune system [11]. Subcortical structures of the central nervous system seemed to be involved in several immunological functions and sepsis [4,10,11]. However, it is unclear how much impairment is present in the humanized mice due to their xenotransplant nature, considering that mice nervous systems will interact with human cells. Additionally, specific organs have a slightly different function between humans and mice. The spleen is an immunologically significant organ in rodents, but its function is somewhat less important in humans. Nevertheless, several researchers report the spleen-based leukocyte alterations in post-septic humanized mice, while examining lymph nodes could be more reflective of changes in human [92]. Furthermore, humanized mice may exhibit impaired interactions between the immune system and organs because several human factors (cytokines) interact with mouse hosts (hormones, organs), even though some other mediators are potentially identical (prostaglandins) [92].

The final limitations to using humanized mice in models of sepsis are the cost and availability of mice. Humanized mice are expensive to procure and to maintain. Recovery of the immune system after grafting the animals with stem cells takes several weeks. During that time, animals must be housed in a sterile facility due to their impaired immunity. Additional expenses at this stage are the grafting and cytokine boosts. Animal attrition may result in significant expenses. Therefore, an essential question concerning researchers is whether all these expenses justify a better approximation of human sepsis. A subsequent concern is the availability of mice; they must be procured locally or obtained from a commercial vendor. Obtaining animals from a local facility has several advantages if there is evidence of provider expertise in manufacturing these animals. Considering significant labor requirements and the length (~3 months) of the process necessary to generate humanized mice, assistance from a local core facility specializing in working with these models is helpful at this stage and should be sought.

### 3.3. Do Humanized Animals Fulfill Their Potential in Advancing the Field of Sepsis?

In 1992, one of the first publications mentioning humanized mice as the model emerged [42]. Since then, humanized mice have been frequently considered, praised, and applauded in numerous reviews as the most promising tools [25,38,43,44,47,78,93]. After all, the logic behind their application is appealing and seemingly sound.

This high level of optimism should be contrasted with the relatively small contribution of humanized mice to the general knowledge of sepsis. Since 1992, there have been fewer than 50 original papers published about sepsis and septic shock including humanized mice. This number is substantially low compared to the higher volume of publications about sepsis in general (*n* = 168,000) or utilizing mice to investigate sepsis (*n* = 13,000). The vast majority of the researchers published one manuscript involving humanized mice without any apparent sequels. The authors often quote high monetary and labor requirements, significant deficit in basic science, and a lack of clear advantages in accelerating drug development as significant reasons to abandon humanized models.

### 3.4. Future of Humanized Animals in Investigation of Septic Shock and Sepsis

Any scientific model’s development should always be linked to the clinical problem it is trying to address in all its complexity. Application of humanized mice to study sepsis encounters an unclear definition of the disease in question, and high heterogeneity [2,5,10,13,16,61]. Furthermore, several confounders related to existence of the human immune system co-existing in mouse hosts must be considered [19,48]. The progress in engineering humanized mice may yield unique experimental conditions considering the dynamism of grafts interacting with host.

One of the reasons for implementing humanized mice is a more effective translation of the therapies into clinical reality. There is an increasing implementation of humanized mice into pre-clinical trials [41,53,54,64,84,85,86,90]. There is also the increasing emphasis on the long-term outcomes of septic patients [1,10]. Unfortunately, most studies euthanized the animals within 24–48 h [20,23,53,92]. The most comprehensive study was performed over 28 days, which mimics the clinical estimation of sepsis mortality [31]. As we know, at the onset of sepsis, the human immune system becomes dysregulated and damages organ function directly or indirectly. Attention to organ failure should be included in research protocols. Some have suggested developing humanized mice with two organs: the immune system and lungs, to mimic certain conditions [78]. Others have suggested supplementing animals with human probiota [64].

In conclusion, humanized models are valuable tools, but they are still in their infancy. Several confounders related to the xenotransplant nature of these animals renders any research to be scrutinized and related to other investigations.

## 4. Materials and Methods

We selected 136 publications from PubMed using keywords such as humanize mice, sepsis, and septic shock. This database was manually reviewed, and 8 reviews were eliminated, while 86 publications were found not relating to humanized mice in terms of stem cell transplantation. We eliminated any manuscript referencing humanized mice in the expression of other human tissues from immune ones. Subsequently, 54 publications are discussed. The representative samples of most critical publication are summarized in Table 2.

## Figures and Tables

**Table 1:** Types of humanized mice.

Type	Grafting	Advantages	Disadvantages
PBMC	Peripheral mononuclear cells	Simple model; graft vs host disease common	Only for short-term experiments, lack of myeloid cells and granulocytes
SRC	CD34 stem cells	Multilineage development of the human immune system	Slow development of the system; relatively immaturity of the human immune system
BLT	Hematopoietic CD34+ cells, thymus, liver	Very robust reconstitution of the human immune system	Prolonged development of the immune system, emergence of graft vs host disease, laborious

**Table 2:** Selected key manuscripts describing the application of humanized mice to study sepsis and infections.

Author	Humanized Animals	Model	Major Finding	Remarks
**Unsinger**	NOD-scid IL2rγ^(null)^ with an adoptive transfer of hCD34^(+)^ hematopoietic cord blood stem cells.	CLP	Sepsis induced marked elevations in human pro- and anti-inflammatory cytokines as well as a dramatic increase in human T and B cell apoptosis	Acute model
**Rodewohl**	NOD-scid gamma mice transplanted with human hCD34^(+)^ stem cells	LPS stimulus with euthanize after 6 h	LPS stimulation induced a decrease in CD14+ monocytes in peripheral blood, an up-regulation of activation markers on different cell subsets such as myeloid dendritic cells, and a release of the human cytokines TNF-α, IL-6 and IL-10.	Significant age difference
**Ye**	NOD/scid/IL2Rγ−/− mice BLT model	CLP	Humanized mice had high serum levels of HMGB1 as well as multiple human, but not murine, proinflammatory cytokines, and uniformly succumbed	
**Libby**	hu-SRC-SCID	Typhoid inoculation	*S. Typhoi* can replicate, causing a lethal infection with pathological and inflammatory cytokine responses resembling human typhoid	
**Tseng**	(SCID)/IL2 γ−/− (NSG) mice engrafted with human CD34+ umbilical cord blood cells	Staph skin inoculation	Humanized mice exhibit larger cutaneous lesions upon infection with PVL+ versus isogenic PVL− *S. aureus*	Granulocytes proved to be a rescue
**Shaler**	NOD-scid IL-2Rgammanu	SEB/Staph exposure	Critical role of MAIT during Staph(+) infection	
**Knop**	NOD.Cg-*Prkdc^scid^ Il2rg^1Wjl^*/SzJ (NSG) CD34 graft	Intraperitoneal Gram-positive sepsis	*S. aureus* infection induced T cell activation, apoptosis, and Fas receptor expression i	
**Laudanski**	NOD-scid γ−/−mice BLT model	CLP model with 28 days follow up	Weight loss and mortality similar to human sepsis. Robust IL-6, M-CSF, and TNF production. Depletion of dendritic cell	
**Lapko**	NOD-scid γ−/−mice BLT model	CLP model with 28 days follow up	Robust M-CSF production and	
**Skierecki**	NOD.Cg-Prkdc/scidIL2rgamma (NSG) mice with the human cord blood CD34(+)	LPS and CLP model	Early changes in bone marrow progenitors in CLP sepsis regulated by Notch	
**Skierecki (2019)**	NOD.Cg-Prkdc/scidIL2rgamma (NSG) mice with the human cord blood CD34(+)	CLP	Sepsis induced a generalized up-regulation of both human and murine plasma cytokines (TNFalpha, IL-6, IL-10, IL-8/KC, MCP-1); it was additionally aggravated in P-DIE vs P-SUR. Human cytokines were strongly overridden by the murine ones (approx. ratio 1:9) but human TNFα was 7-fold higher than mouse TNFα	The effect of host environment on human leukocytes was attributed to increased mortality
**Ernst**	Humanized mice	*E. coli* infection	Leukocyte trafficking to the site of infection	Positive effect of indomethacin and steroid on cytokine profile
**Szabo**	Humanized mice	Systemic exposure to SEB/ toxic shock syndrome	Rapid accumulation of granulocytic myeloid derived suppressor cells	
**Zhao**	Humanized mice	CLP model of sepsis	antoPD-L1 treatment improved survival	
**Mota**	NOD-scid IL2rgamma(null) mice received an adoptive transfer of hCD34(+) cord blood stem cells.	Dengue inoculation	Emergence of the rash, fever, and thrombocytopenia	
**Kuruvilla**	RAG2(−/−)gamma(c)(−/−) mice were xenografted with human CD34+ stem cells	Dengue inoculation	Emergence of anti-Dengue IgG neutralizing antibodies for 6 weeks	
**Costa**	NOD-scid-IL-2Rγnull (NSG) mice grafted with CD34+ stem cells	Dengue inoculation	Critical role of NK cells and IFNg in controlling the infection	
**Rodriguez**	NOD.Cg-*Prkdc^scid^Il2rg^tm1Wjl^*Tg(HLA-A2.1)1Enge/SzJ (NSG-A2) grafted with CD34	Intravenous adenovirus injections	Development of acute and persistent adenovirus infection	
**Melkus**	NOD/scid BLT model	Administration of TSST-1	Expansion of human Vbeta2+ T cells, release of human proinflammatory cytokines and localized, specific activation and maturation of human CD11c+ dendritic cells	
**Jarman**	hu-PBL-scid mice	Immunization with IgE	Critical role of antigen-presenting cells; role of spleen in generating immune system response	
**Vaughan**	neonatal NOD/scid/IL2R null transferred with PB		Emergence of immature MO with reduction in immunostimulatory T cell capacity	
**Lim**	NOD-scid mice reconstituted with PDC-depleted peripheral blood mononuclear cells	Implantation of EBV-positive graft	Production of IFNg by EBV-positive cells	

## Data Availability

Not applicable.
